# Effect of Electromagnetic Stirring on the Microstructure and Properties of Fe-Cr-Co Steel

**DOI:** 10.3390/ma11081437

**Published:** 2018-08-14

**Authors:** Lin Zhang, Yuhang Hou, Xiao Guo, Zhaolong Xiang, Engang Wang

**Affiliations:** Key Laboratory of Electromagnetic Processing of Materials (Ministry of Education), Northeastern University, Wenhua Road, Shenyang 110819, China; hyh910658860@outlook.com (Y.H.); 13322463877@163.com (X.G.); xzl5612@163.com (Z.X.)

**Keywords:** high chromium steel, electromagnetic stirring, microstructure, hardness, tensile strength

## Abstract

High chromium steel has been synthesized by an induction furnace adopting electromagnetic stirring (EMS). Varying amounts of cobalt was added to obtain 3, 6, and 12% Co in the steel. The melt was allowed to solidify with or without EMS in a rotary magnetic field. The effects of the varying cobalt content and the stirring have been characterized by the microstructural evolution and the consequent improvement in mechanical properties. The application of a rotary EMS during solidification has shown a significant effect on the grain refining, the reduction of element segregation, the promotion of eutectic volume fraction, and the consequent improvement of mechanical properties, including hardness and high-temperature strength. The formation mechanism of the eutectic structure and the precipitation of M_7_C_3_ and M_23_C_6_ carbides was discussed according to the calculated phase diagram. The increment of cobalt content improved the eutectic volume fraction. Cobalt addition also enhanced the hardness and the yield tensile strength, provided that the ingot structure was homogenized by the EMS.

## 1. Introduction

High chromium steel is characterized by a good mechanical performance at an elevated temperature, which is generally used in a variety of engineering applications, such as the manufacturing of components for advanced power plants. The mechanical properties of high chromium steel were achieved by the alloying elements that provided solution and dispersion strengthening. Cobalt is an important element used in steels to increase the resistance to high temperature [1,2]. Cobalt retards the diffusion by raising the Curie temperature, and inhibits the coarsening of carbides [3]. In martensitic creep resistant steels, the precipitation strengthening of Laves phase and M_23_C_6_ dropped as the degree of cobalt alloying decreased [4]. In high chromium heat-resistant steels, cobalt addition improved the volume fraction of the Laves phase [5,6]. Adding cobalt to steel suppressed the δ-ferrite formation in the normalizing process of the high chromium steels, and improved the creep properties at elevated temperatures [7,8]. In this work, we investigated the microstructure and properties of Fe-Cr-Co steels. We used electromagnetic stirring (EMS) to reduce the element segregation of chromium or cobalt.

In the solidification of metals and alloys, forced stirring can be used to improve the microstructure and performance of the ingots. The forced vibration or convection is created in the melt by a mechanical, ultrasonic or electromagnetic field. The EMS is a non-contact mode of stirring, which avoids the contamination of melt, and also avoids damage to the agitator blade or the ultrasonic probe used in other stirring methods. EMS has been widely applied to the casting of metals, and has a series of effects to improve the quality of ingots. First, electromagnetically-induced flow causes grain refinement and enhances the ratio of equiaxed grain. Fragmentation of the dendrite, and heterogeneous nucleation are the probable causes for the promotion of the columnar-equiaxed transition. EMS has been reported to promote equiaxed grain and reduce the average grain size in alloys such as various steels [9,10,11], super alloys [12,13], copper alloys [14,15], magnesium alloys [16,17], and aluminum alloys [18,19,20]. Microstructure refinement is helpful to improve the mechanical properties and characteristics of the metal alloys.

Besides the effect of grain refinement, studies indicated that the EMS could influence many aspects of solidification. Strong stirring resulted in the removal of bulk liquid superheating. and lead to a relatively cold liquid on the solidification front [18], reduced casting defects such as porosity, and improved the surface quality [21,22]. EMS has also been reported to remove inclusion in melts, and improve cleanliness, such as in steels [23,24,25], and Incoloy 825 alloys [12]. Moreover, EMS was used to eliminate the micro- and macrosegregation of solute elements in ingots [26,27]. Also, the EMS applied to the solidification of metals led to the refinement and better distribution of precipitates [28,29].

In this work, the effect of cobalt addition was studied in detail using three kinds of steels, with each of their as-cast ingots being compared in terms of microstructure and mechanical properties. The effect of EMS during the solidification process was also investigated. The comparison of grain size, eutectic structure, and the change in hardness and high temperature strength was discussed.

## 2. Materials and Methods

In this study, a high chromium steel was used, which contained 18% Cr, 2% Ni, 1% Mo (wt %). Besides, cobalt of 3, 6, and 12% were added to the specimens. In addition to the basic alloying elements, the steels also contained 0.2% C, 0.2% Mn, and 0.1% Si.

**Figure 1:**
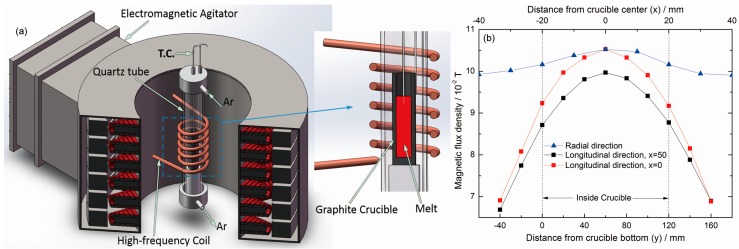
This is a schematic diagram of the experimental set-up (**a**) and the dismatribution of the max magnetic flux density (**b**).

The experimental set-up (Figure 1a) for a rotary EMS constituted an electromagnet with an inner diameter of 280 mm and a height of 300 mm, and a crucible was placed inside a copper coil at the center of the electromagnet to melt the steel inside it. The distribution of the max magnetic flux density is shown in Figure 1b. A quartz tube sealed by flange was placed outside the crucible, and high purity argon was introduced into the tube. The crucible was combined with an inner part of alumina and an outer part of graphite, with an inner diameter of 40 mm and a height of 150 mm. The crucible was protected by the high purity argon.

The steel was melted by induction heating, and the temperature was raised to 1600 °C. The melts were allowed to solidify with or without the EMS in a rotary magnetic field generated by a current set at 8 Hz/300 A. The ingot was a cylindrical shape, and had dimensions of diameter 40 mm and length 120 mm. Each ingot was subsequently sectioned longitudinally and etched to reveal the microstructure. The macrostructures and microstructures of the samples were observed by optical microscopy and the scanning electron microscopy (SEM) respectively. The optical microstructure was observed under a LeicaDMI 5000M microscope (Leica Microsystems, Wetzlar, Germany). The SEM microstructures were observed using an Ultra Plus FESEM scanning electron microscope (FEI Technologies Inc., Hillsboro, OR, USA). The phases were identified by the X-ray diffraction analysis using a Philips X’Pert Pro MPD diffractometer (PANalytical Co., Almelo, the Netherlands). Image-Pro Plus software (Version 6.0, Media Cybernetics, Inc., Rockville, MD, USA) was used to analyze the size and the area fraction of phases.

In order to evaluate the mechanical performance of the samples, tensile strength and hardness tests were performed respectively. Hardness measurements were taken from a polished but unetched sample, using a Vickers hardness tester, with a load of 196 N and loading time of 15 s. Tensile tests were performed in a Shimadzu AG-X 100 kN testing machine (Shimadzu Corp., Kyoto, Japan) in accordance with the requirements and recommendations of the ISO 6892-2:2011 [30] for elevated temperature. The high temperature tensile tests were performed at a velocity of 1 mm/min and a temperature of 600 °C.

## 3. Results and Discussion

### 3.1. Casting Microstructure

**Figure 2:**
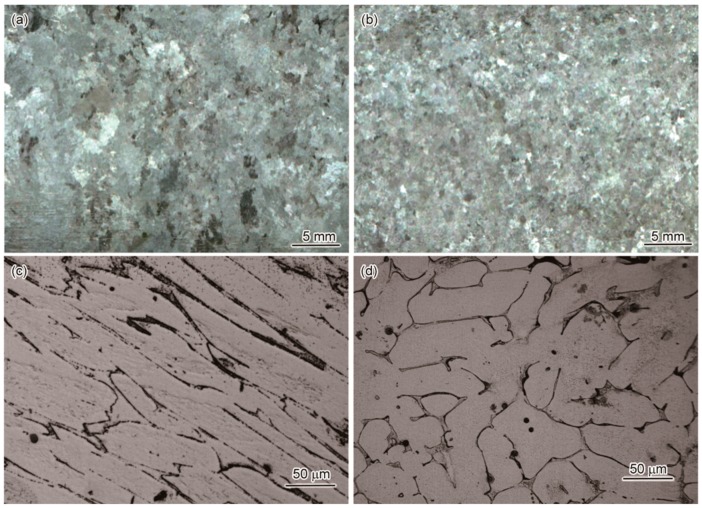
Morphology of grains in cast Fe-18%Cr-3%Co steel without stirring (**a**,**c**), and with electronmagnetic stirring (EMS) generated by current set at 8 Hz/300 A (**b**,**d**).

The effect of EMS on both the macrostructure and microstructure of Fe-Cr-Co steels was investigated. Several typical macrostructures obtained by the experiment without and with stirring are shown in Figure 2a,b respectively. As revealed by the figures, the effect of stirring on the grain refinement was very much prominent. Compared with the structure observed in an as-cast steel, the grains became finer when the EMS was imposed. This refinement was observed in all the three steels used. As revealed by the former researchers [9,10,11,12], the grain refinement was attributed to the strong melt convection generated by the EMS that promoted the transition from the columnar dendrite to the equiaxed dendrite. The strong stirring caused a dendrite fracture, and the dendrites were broken into small pieces. Then these fragments moved into the other regions of the melt to promote the nucleation of new dendrites as the nucleation sites. Thus, EMS increased the nucleation rate and enhanced the amount of equiaxed dendrites.

**Figure 3:**
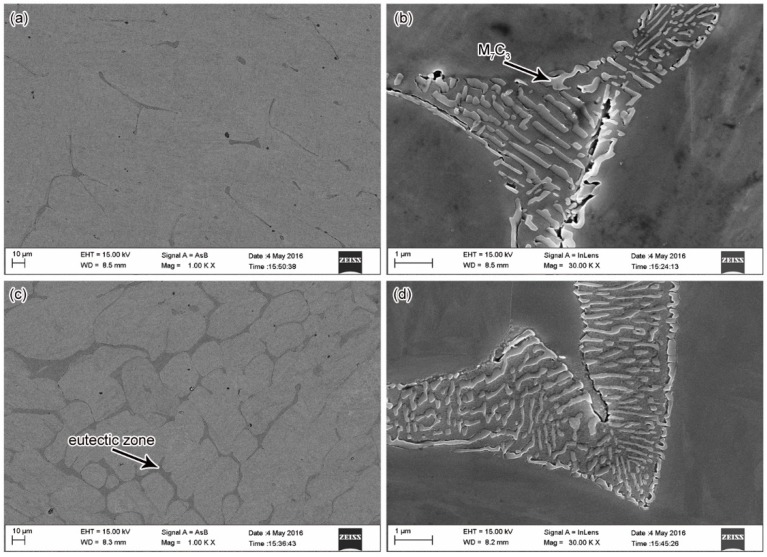

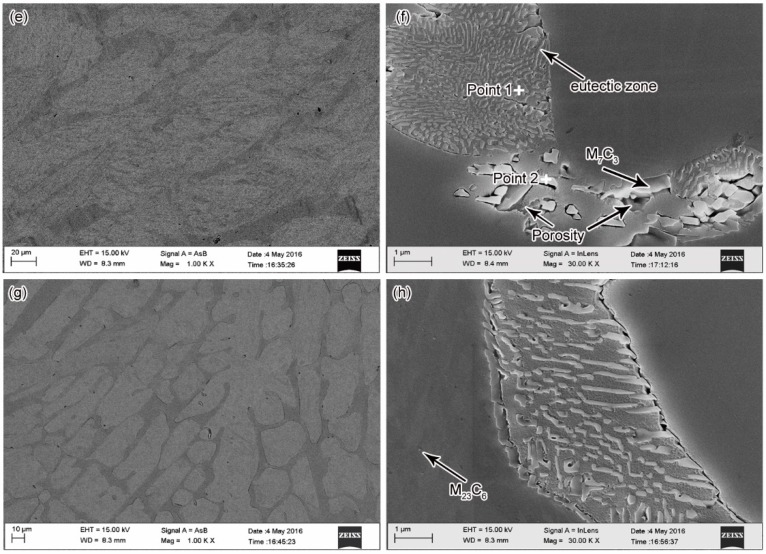
Field emission scanning electron microscopy (FESEM) micrographs of steel with and without EMS: (**a**,**b**) Fe-18%Cr-3%Co, without stirring; (**c**,**d**) Fe-18%Cr-3%Co, stirred at 8 Hz/300 A; (**i**,**j**) Fe-18%Cr-12%Co, without stirring; (**k**,**l**) Fe-18%Cr-12%Co, stirred at 8 Hz/300 A.

The microstructures of the 3% Co steel cast without and with stirring are shown in Figure 2c,d respectively. These were taken from the center of the ingot mid-plane, 60 mm from the ingot base. Columnar grains were observed in the sample without EMS, which grew parallel to the direction of heat flow, whereas the EMS sample only comprised of equiaxed grains. The grain boundaries tended to be more clear and continuous in the case of EMS. To investigate this in detail, we observed the morphology of grain boundaries using field emission scanning electron microscopy (FESEM). The micrographs of the solidified steel with and without EMS are shown in Figure 3, in which Figure 3a,c,e,g on the left side represent the backscattered electron images of the steel with different cobalt content. The backscattered electron images enhanced the averaged atomic number contrast for observation of the shape of carbon-rich regions. There were eutectics existing at the grain boundaries that contained carbides, due to non-equilibrium solidification. Therefore, the shapes of the eutectic and the grain boundaries were clearly observed. Although the detailed morphology inside the eutectic region could not be detected with the backscattered electron images, a clear eutectic structure with fine carbide precipitates was observed in a highly magnified secondary electron image, as exemplified in Figure 3b,d,f,h.

**Figure 4:**
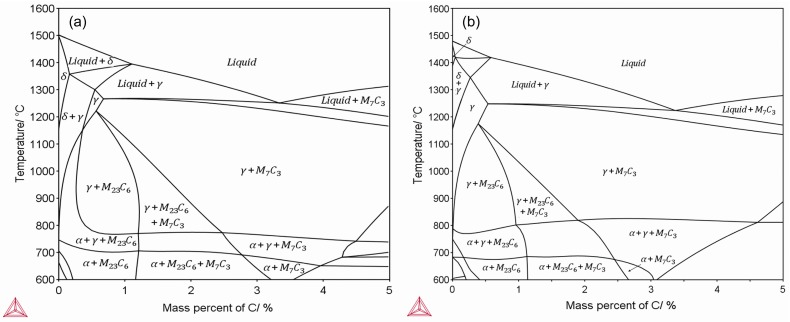
Phase diagram calculated by Thermo-Calc: (**a**) Fe-18%Cr-3%Co and (**b**) Fe-18%Cr-12%Co.

**Figure 5:**
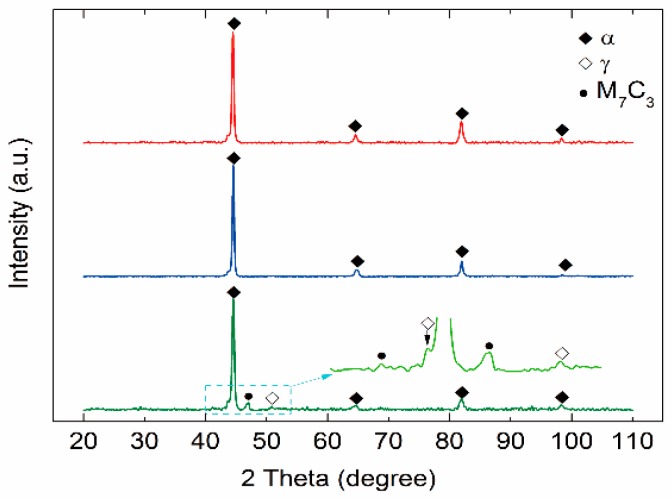
X-ray diffraction (XRD) pattern of steel samples with different cobalt contents: (**a**) Fe-18%Cr-3%Co; (**b**)Fe-18%Cr-6%Co; (**c**) Fe-18%Cr-12%Co.

To identify the phases shown in Figure 3 and to understand the solidification process of Fe-Cr-Co steel, we calculated the phase diagrams of the Fe-18%Cr-3%Co steel and the Fe-18%Cr-12%Co steel, with the diagrams shown in Figure 4. According to the phase diagram, the bcc *δ* phase first nucleated in the melt and grew, then the fcc *γ* phase grew in the next turn, and the original δ phase also changed into the *γ* phase. During the growth process of the *δ* phase and the *γ* phase, the solidifying front pushed the rest of the liquid into the boundary of dendrites, and some elements, including carbon, tended to be enriched in the residual melt. In the end, the residual melt reached a eutectic concentration. The residual liquid then decomposed via a eutectic reaction, into the M_7_C_3_ carbides and the austenite (*γ*). The austenite changed into ferrite as the temperature dropped. As revealed by the phase diagrams, the matrix phase of the steels was bcc ferrite (*α*), which corresponded to the matrix grains in Figure 3. An X-ray diffraction (XRD) pattern shown in Figure 5 also proved that the matrix was a fcc *α* phase. The phase diagrams revealed that the eutectic of the steel contained M_7_C_3_ carbides, and there should be M_23_C_6_ carbides precipitated at low temperature. Hence, the eutectic carbides shown in Figure 3b,d,f,h were M_7_C_3_ carbides, and the eutectic consisted of ferrite + M_7_C_3_. In Figure 3h, some fine precipitations were observed in the matrix, which were probably M_23_C_6_ carbides.

Figure 3a,c provide the 3%Co as-cast steel without and with stirring, respectively. In the steel solidified without stirring, the eutectic region occupied 2.32% of the section area. With the application of EMS, the area fraction of the eutectic region was increased to 21.7%. Without the EMS, the eutectic was short and discontinuous, whereas it turned out to be long and continuous when the EMS was applied. There was also an increase in the average width of the eutectic region from 2.5 µm to 5.3 µm, accompanying the application of EMS. The eutectic formed on the grain boundaries and connected with each other, which made the grain boundary more complete and clear. As discussed above, there was a prominent effect of grain refinement by the EMS. The average grain sizes without and with EMS were 52.4 µm and 26.5 µm respectively. The eutectic was distributed dispersively with an increasing amount of grain boundaries.

It is apparent that the cobalt content also influenced the eutectic proportion in the section area. As shown in Figure 3g, the 12%Co as-cast steel with the stirring contained eutectic that occupied 33.93% of the section area, which was 12.23% higher compared with the 3%Co steel shown in Figure 3c. As revealed by the phase diagram shown in Figure 4, when the cobalt content increased from 3% to 12%, the region of *γ* phase was enlarged and moved to the left side; moreover, the region of the liquid + *γ* phase also moved to the left, indicating that the proportion of residual liquid was enhanced for a given composition and temperature in this region. The volume of the eutectic region increased with increasing residual liquid, which was consistent with the experimental results shown in Figure 3. The XRD patterns shown in Figure 5 also indicated that the proportion of *γ* phase and M_7_C_3_ carbides in the 12%Co steel was higher than that in the 3%Co and 6%Co steels.

Figure 3e provides a coarse grain structure with some thick eutectic regions. Although the grain boundaries appeared to be dark due to the existence of carbide, there were some dark pieces that extended into the ferrite grain, indicating that some carbides or small pieces of eutectic regions existed inside the ferrite grain, that led to an unclear morphology of the grain structure in the backscattered electron image. A highly magnified secondary electron image shown in Figure 3f revealed that there were two kinds of eutectic regions with different carbide sizes; one had an average width of 0.27 µm, and the other had an average width of 0.07 µm. During the solidification without stirring, the concentration of elements tended to be inhomogeneous in the residual melt, and this led to the different rates of constitutional undercooling in each region, which in turn caused the different eutectic carbide sizes. As shown in Figure 3b,f, the large carbides tended to reduce the bonds between the grains and caused porosities that could lead to the fracture of the steel. As shown in Figure 3d,h, the carbides in the stirred samples had a smaller size discrepancy, and the porosities were much smaller compared with that without stirring.

**Figure 6:**
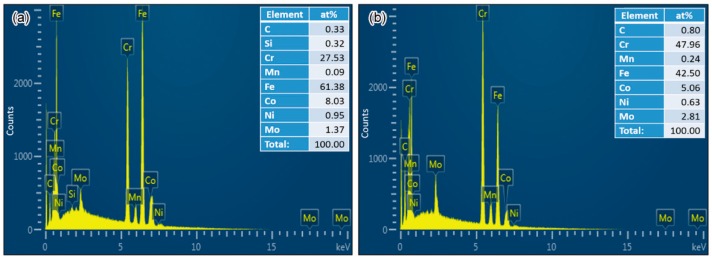
Comparison of the energy-dispersive X-ray spectroscopy (EDS) results of the point 1 (**a**) and point 2 (**b**) in Figure 3f.

We identified the concentrations of alloying elements in the two eutectic regions with different carbide sizes shown in Figure 3f, using energy-dispersive X-ray spectroscopy (EDS); the results are shown in Figure 6. The carbide size was much smaller than the EDS detect zone; therefore, these results reflected the composition of the eutectic regions, including both the ferrite matrix and the carbides. Compared with the nominal composition of the steel, chromium tended to segregate to the eutectic region along the grain boundary. The eutectic with large carbides had a chromium concentration of 47.96%, which was nearly 20% higher than that in a nearby eutectic region with small carbides. On the other hand, the Fe concentration decreased from 61.38% to 42.5%. The discrepancy in the element concentration of the different residual melts was a probable cause of the different eutectic carbide sizes.

**Figure 7:**
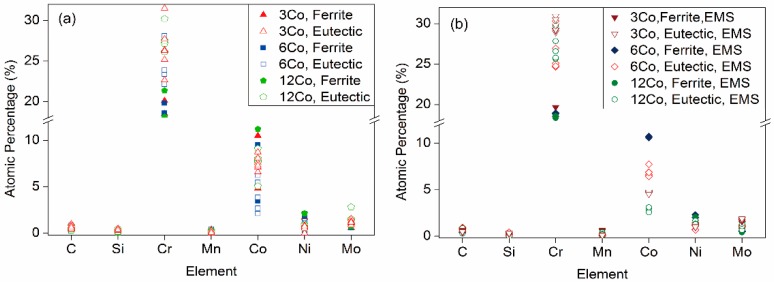
Concentrations of the alloying elements analyzed by EDS: (**a**) without EMS; (**b**) with EMS.

We also analyzed the concentrations of alloying elements using EDS in a series of eutectic regions and ferrite grains in the steels of different solidification conditions, and the result is shown in Figure 7. It should be mentioned that the EDS provides a semi-quantitative result, which is especially inaccurate for light elements such as carbon. The atomic percentage of different elements obtained from the ingots were found to have a number of common features, typified by the data shown in Figure 7. The contents of Si and Mn were similar in each condition. However, chromium and cobalt both had a large deviation in different regions during solidification; they are the two main elements that provide hardening to the steel, and the deviation of their concentration should have an impact on the mechanical properties. As revealed by Figure 7, ferrite tended to contain more cobalt and nickel than eutectic, whereas it lacked chromium and molybdenum at the same time. The eutectic region formed by the residual melt was rich in the elements of chromium and molybdenum. The ferrite grains had a steadier composition compared with the eutectic region, and the composition fluctuation in the eutectic region mainly focused on chromium and the cobalt. With EMS, the concentration fluctuation of chromium and cobalt both decreased compared to that without stirring.

### 3.2. Mechanical Properties

**Figure 8:**
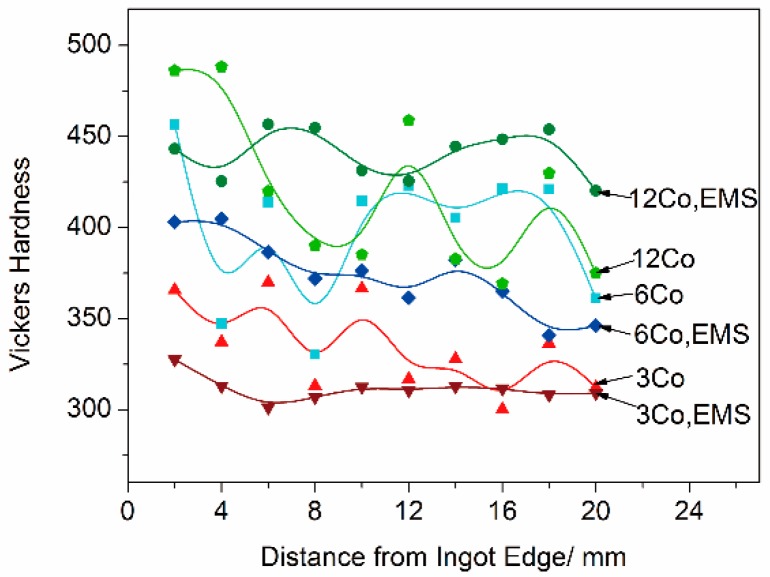
Hardness change with distance from ingot edge.

Figure 8 shows the hardness change with distance from the ingot edge for the steels with different cobalt contents, with and without stirring. The values of hardness had a trend to decrease with increasing distance, indicating that the ingot edge was harder than the center. Since cobalt was a main strengthening element in the steel, the hardness was also enhanced with increasing cobalt content. All of the stirred samples had a steadier trend of hardness compared with the samples without stirring. A possible explanation for this discrepancy in hardness distribution was the different feature of the microstructure that was discussed above. The hardness of both matrix and carbide phases determined the hardness of the steel. The ferrite grain size and the distribution of the carbides strongly affected the mechanical properties of the steel. As discussed above, the samples with stirring had a finer grain structure and a more dispersive distribution of eutectic regions, compared with the samples without stirring. Thus, the distribution of carbides phase was dispersive and homogeneous, which contributed to the steady distribution of hardness.

**Figure 9:**
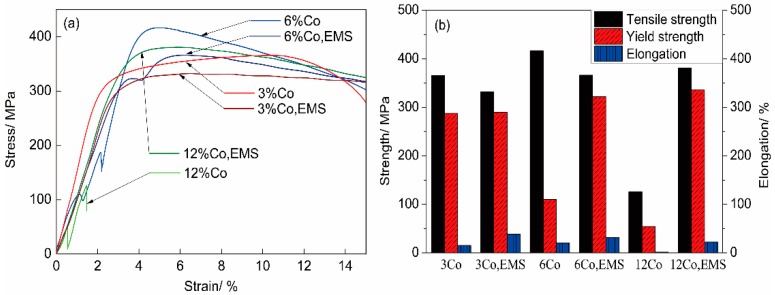
Tensile test results at a high temperature of 600 °C: (**a**) Stress-strain curve; (**b**) tensile strength, yield strength, and elongation.

The results of tensile strength at an elevated temperature of 600 °C are provided in Figure 9. As shown in Figure 9a, the stress-strain curve was smooth for the 3%Co steels, indicating a continuous yield without a distinct yield point. However, there were yielding points that were present in the 6%Co steels, indicating a discontinuous yielding. One yielding point presented for the steel with EMS, and two yielding point presented for the steel without stirring. In the curve of the 12%Co steel without stirring, the first yielding point occurred at a low stress, then it broke at the second yielding. In contrast, the curve showed a continuous yielding for the 12%Co steel with EMS. This discrepancy could be explained by the microstructures and the element distribution. With stirring, the refinement of grain size had a positive impact on the improvement of strength and plasticity, which contributed to the increment in the tensile strength and the elongation-to-fracture. The steels without stirring had a relatively coarse microstructure (as shown in Figure 3) and serious element segregation (as shown in Figure 7). The grain boundary, the eutectic, and the carbides provided strengthening to the steel. Besides, small interstitial atoms clustered around the dislocations interfered with the slip and raised the yield point. As discussed above, there could be two kinds of eutectic with different carbide sizes and different element concentrations in the steels without stirring, they could offer different strengthening effects to the steel. Especially in the 12%Co steel, there were large porosities caused by large M_7_C_3_ carbides (as shown in Figure 3f), which could lead to the fracture of the steel with small stress. These factors may explain the yielding behavior of the steel without stirring. As shown in Figure 9b, although the highest tensile strength 416.65 MPa was present for the 6%Co steel without stirring, it had a relatively low yield strength of 110.19 MPa. The highest yield strength of 335.58 MPa was from the 12%Co steel with EMS. The stirred steels had a higher yield strength and a higher elongation compared with that without stirring, and the highest elongation rate was 38.87% from the 3%Co steel with stirring. Comparing all the stirred samples that have a homogenous structure, there was a steady trend that both tensile strength and yield strength increased with increasing cobalt content.

## 4. Conclusions

The effect of EMS on the microstructure and mechanical properties of high chromium steel was experimentally investigated.

The steel matrix consisted of ferrite grains, and the eutectic region was distributed along the grain boundary. By applying EMS, the grain structure was refined, leading to a uniform distribution of the grain boundary. The eutectic region consisted of M_7_C_3_ carbides and the ferrite matrix. The volume fraction of the eutectic was promoted by both the cobalt increment and the EMS, which in turn resulted in a dispersive distribution of carbides. The effect of cobalt increment on the promotion of eutectic was caused by the position and shape change of the liquid + *γ* phase region in the phase diagram. There was a distinct element segregation of cobalt and chromium between the ferrite and the eutectic region, which were both reduced by stirring.

With the decrease of element segregation and the refinement of microstructure, the distribution of hardness was steady in the steels with stirring, whereas it fluctuated frequently in the steels without stirring. Hardness also had a trend to increase with increasing cobalt content. The increasing cobalt content up to 6% contributed to the discontinuous deformation in the stress-strain curve, and this caused a considerable decrease of strength when the cobalt content reached 12%. The application of EMS increased the yield strength and the elongation rate of the steels; it also reduced the adverse effect caused by the high cobalt percentage, and thus, both the tensile strength and the yield strength increased with increasing cobalt content.
